# Giant solid pseudopapillary neoplasm of the pancreas in an adolescent girl: a case report with narrative review

**DOI:** 10.3389/fonc.2026.1871126

**Published:** 2026-07-15

**Authors:** Hang Zhou, Jinqin Zha, Yu Yang, Yiwei Hou, Chongyuan Chen, Mingzheng Hu, Rongchun Xing

**Affiliations:** 1 The First College of Clinical Medical Science, China Three Gorges University, Yichang, Hubei, China; 2 Department of Hepatobiliary Surgery, Yichang Central People’s Hospital, Yichang, Hubei, China; 3 Department of General Practice, Yichang Central People’s Hospital, Yichang, Hubei, China; 4 Department of Endocrinology, Yichang Central People’s Hospital, Yichang, Hubei, China

**Keywords:** accessory spleen, case report, distal pancreatectomy, laparoscopy, pancreas, solid pseudopapillary neoplasm, splenectomy

## Abstract

Solid pseudopapillary neoplasm is a rare epithelial tumor of the pancreas with low malignant potential and a marked predilection for adolescent girls and young women. Although the overall prognosis is favorable after complete resection, preoperative characterization and surgical planning become more challenging in giant tumors with intratumoral degeneration, hemorrhage, and adjacent vascular compression. We report a 15-year-old girl who presented with acute abdominal pain persisting for 24 hours after strenuous physical activity. Imaging revealed a giant mixed solid-cystic mass in the pancreatic body and tail, and computed tomography angiography and venography demonstrated narrowing of the splenic vein with collateral venous circulation. After multidisciplinary assessment of oncologic safety and perioperative bleeding risk, the patient underwent laparoscopic distal pancreatectomy with splenectomy. Histopathology confirmed solid pseudopapillary neoplasm with negative margins and no nodal metastasis; ectopic splenic tissue was identified in the peripancreatic fat. Immunohistochemistry showed nuclear/cytoplasmic positivity for beta-catenin, loss of E-cadherin, positivity for CD10 and CD56, and partial positivity for lymphoid enhancer-binding factor 1, supporting the diagnosis. The postoperative course was uneventful, and no evidence of recurrence was detected on short-term follow-up. This case suggests that giant solid pseudopapillary neoplasm may have been related to acute abdominal pain in the setting of intratumoral degeneration, hemorrhagic change, and local tension effect. Surgical strategy should balance oncologic safety, bleeding risk, and organ preservation. In patients requiring splenectomy, long-term infection prevention should include immunization planning, fever emergency counseling, and individualized antibiotic prophylaxis according to local guidance and risk stratification.

## 1 Introduction

Solid pseudopapillary neoplasm (SPN) is classified by the World Health Organization (WHO) as a pancreatic epithelial neoplasm with low malignant potential (1). It is uncommon among pancreatic tumors and shows a striking predilection for adolescent girls and young women (2–4). Clinically, SPN often grows slowly and may remain silent until it becomes large enough to cause abdominal discomfort, abdominal pain, a palpable mass, or compression of adjacent organs (2–4). Radiologically, it typically appears as a well-circumscribed pancreatic lesion with variable solid and cystic components, hemorrhagic degeneration, necrosis, and capsule-like features; these findings are particularly useful when SPN needs to be distinguished from pancreatic neuroendocrine tumor (PanNET), mucinous cystic neoplasm (MCN), pancreatoblastoma, or accessory spleen-related lesions (4, 5). Although complete surgical resection usually provides an excellent long-term outcome, giant tumors may distort the pancreatic body-tail anatomy, compress splenic vessels, form collateral venous circulation, and increase the technical difficulty of spleen-preserving distal pancreatectomy (3–5). Therefore, perioperative decision-making in giant SPN remains clinically valuable, especially in adolescent patients.

Here, we present a giant SPN in the pancreatic body and tail of a 15-year-old girl. By integrating the perioperative imaging findings, operative decision-making, histopathology, immunohistochemistry, and the current literature, we aimed to illustrate the diagnostic and surgical considerations for giant SPN in an adolescent patient. Particular attention is given to differential diagnosis, preservation versus resection of the spleen, postoperative surveillance, and infection prevention after splenectomy.

## 2 Case presentation

### 2.1 General information

A 15-year-old girl was admitted with abdominal pain persisting for 24 hours. The pain developed after strenuous physical activity and was described as intermittent dull abdominal pain. She had no nausea, vomiting, chest discomfort, or palpitations. An abdominal computed tomography (CT) performed at an outside hospital revealed a large intra-abdominal mass of uncertain nature. On admission, her temperature was 36.5 °C, pulse 93 beats/min, respiratory rate 20 breaths/min, and blood pressure 105/68 mmHg. Physical examination showed mild upper abdominal tenderness without rebound tenderness or a clearly palpable mass. Her past medical, surgical, and family histories were unremarkable.

### 2.2 Laboratory examination

Routine blood tests, liver and renal function tests, coagulation parameters, and tumor markers were within normal limits: alanine aminotransferase (ALT) 7 IU/L, aspartate aminotransferase (AST) 18 IU/L, total bilirubin 7.6 μmol/L, direct bilirubin 2.4 μmol/L, prothrombin time (PT) 14.1 s, international normalized ratio (INR) 1.10, alpha-fetoprotein (AFP) 1.5 ng/mL, carbohydrate antigen 19-9 (CA19-9) 31.5 U/mL, carbohydrate antigen 72-4 (CA72-4) 0.9 IU/mL, and carcinoembryonic antigen (CEA) 0.8 ng/mL.

### 2.3 Imaging examinations

**Figure 1:**
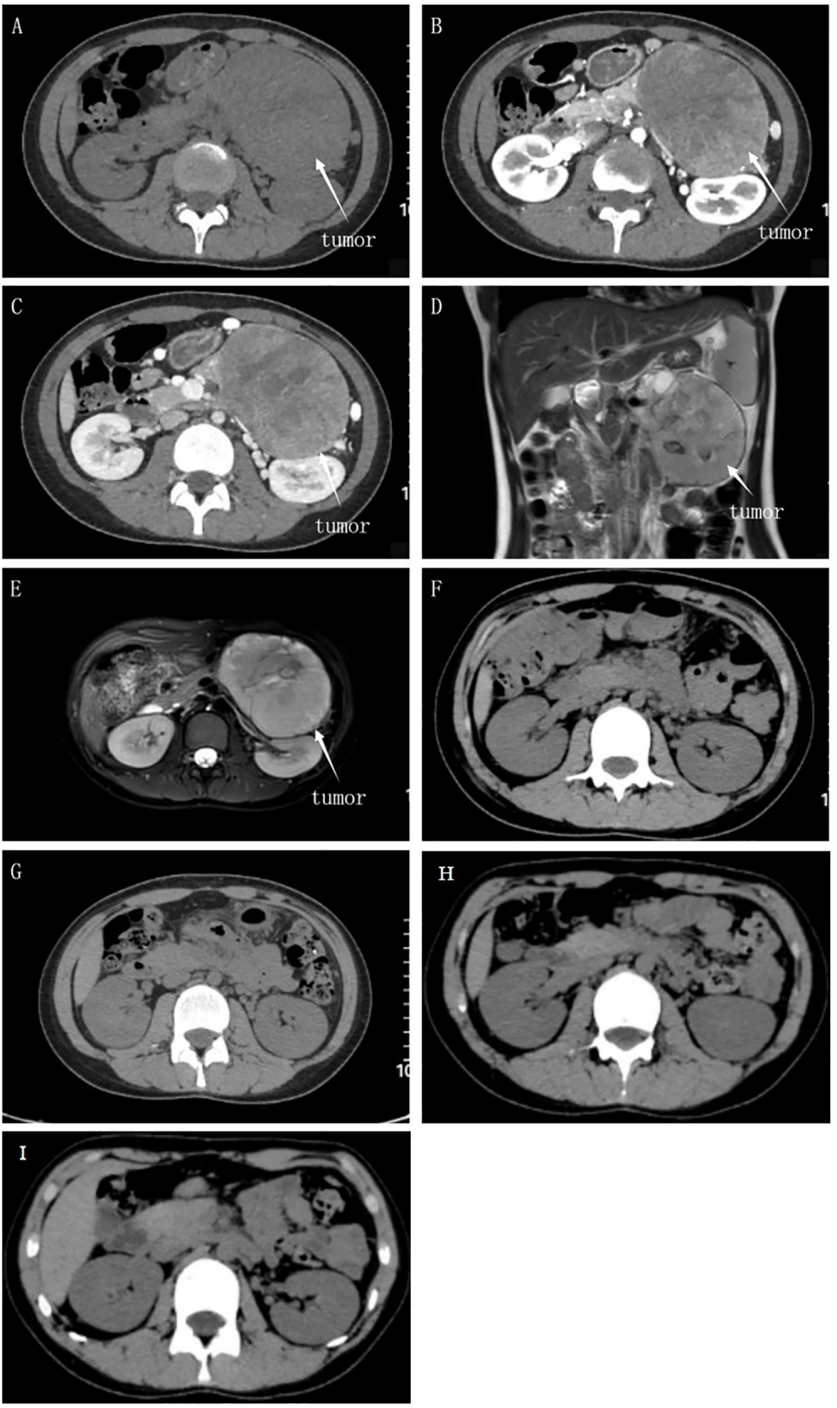
Imaging evaluation and postoperative follow-up. **(A)** Plain CT showing a giant soft-tissue mass in the pancreatic tail region. **(B, C)** Heterogeneous enhancement on arterial and portal venous phase CT. **(D)** Coronal MRI demonstrating upward displacement of the stomach by the mass. **(E)** Axial MRI showing mixed solid-cystic architecture and a capsule-like low-signal rim. **(F)** Postoperative plain CT showing disappearance of the original lesion. **(G)** Postoperative contrast-enhanced CT showing satisfactory recovery of the operative field without abnormal enhancement. **(H, I)** Plain CT at the approximately 6-month follow-up showing postoperative changes after distal pancreatectomy and splenectomy, without a definite recurrent mass.

Abdominal CT at the outside hospital showed a large abdominal mass of uncertain origin. Subsequent computed tomography angiography/venography (CTA/CTV) demonstrated a round mixed low-density lesion in the pancreatic body and tail region measuring approximately 9.9 cm × 7.7 cm, with heterogeneous enhancement and displacement of the stomach, spleen, and left kidney. The splenic vein was focally narrowed, and collateral venous communication was present. Contrast-enhanced magnetic resonance imaging (MRI) revealed a mixed-signal mass with predominantly low T1 signal, patchy hyperintense foci, heterogeneous mildly high T2 signal, focal cystic degeneration, and progressive heterogeneous enhancement of the solid component, suggesting SPN (**Figure 1**). Mild discrepancies in tumor size across examinations were considered explainable by different imaging planes, phases, intraoperative estimation, and specimen measurement.

### 2.4 Preoperative diagnosis and differential diagnosis

Based on the patient’s demographic profile, acute abdominal symptoms, normal tumor-marker profile, and imaging findings of a giant mixed solid-cystic lesion arising from the pancreatic body and tail, the multidisciplinary team considered SPN as the leading preoperative diagnosis. The working differential diagnoses included pancreatic neuroendocrine tumor, pancreatoblastoma, mucinous cystic neoplasm, and an accessory spleen-related lesion of the pancreatic tail. Preoperative imaging showed displacement rather than definite invasion of the stomach, spleen, and left kidney. No radiologic evidence of distant metastasis was identified. Because the lesion was large and closely related to the splenic vessels, the operative plan prioritized complete tumor removal, avoidance of tumor rupture, and safe control of potential bleeding. The definitive diagnosis was reserved for postoperative histopathological and immunohistochemical evaluation.

### 2.5 Surgical treatment

The patient underwent laparoscopic exploration. A giant tumor measuring approximately 10.0 cm × 8.0 cm was found in the pancreatic body and tail. During distal pancreatectomy, the tumor proved densely adherent to the splenic vessels, and further dissection carried a substantial bleeding risk. Because preoperative CTA/CTV had already shown splenic vein narrowing with collateral circulation, spleen preservation was judged unsafe and oncologically less reliable. Laparoscopic distal pancreatectomy with splenectomy was therefore performed.

### 2.6 Pathological findings

Gross examination showed a pancreatic nodular mass measuring 11 cm × 8.5 cm × 5 cm, with a gray-red cut surface, mixed solid-cystic appearance, and focal necrosis. The spleen showed no tumor involvement. A small gray-brown nodule measuring 0.6 cm was present in the surrounding fatty tissue. Histopathology confirmed solid pseudopapillary neoplasm of the pancreas with negative surgical margins. One group 8 lymph node was negative for metastasis (0/1). Well-differentiated splenic tissue was identified in the peripancreatic fat, consistent with a peripancreatic accessory/ectopic spleen.

**Figure 2:**
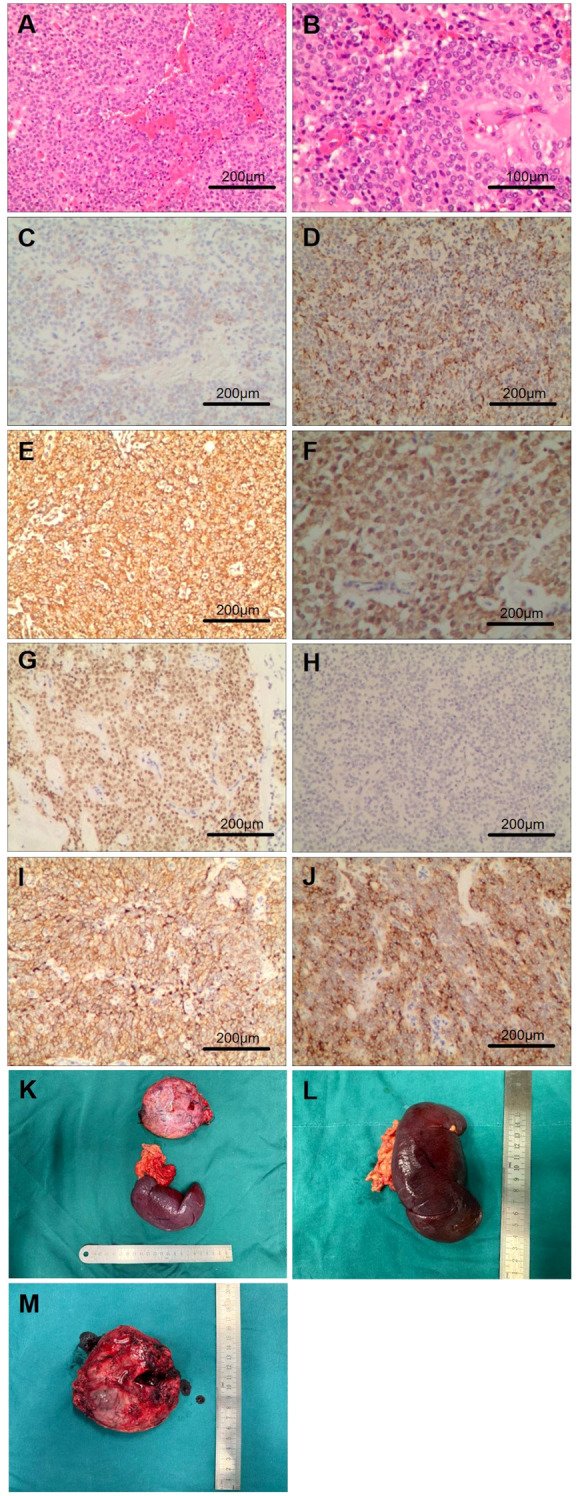
Histopathology and immunohistochemistry of the solid pseudopapillary neoplasm. **(A, B)** Hematoxylin and eosin staining demonstrated solid and pseudopapillary tumor architecture, with hemorrhagic and degenerative changes. Tumor cells were relatively uniform, with round-to-oval nuclei and eosinophilic cytoplasm. **(A)**, ×100; **(B)**, ×200. **(C)** Scattered Syn positivity, ×100. **(D)** Weak focal PCK positivity, ×100. **(E)** Strong vimentin positivity, ×100. **(F)** Nuclear and cytoplasmic β-catenin positivity, ×100. **(G)** LEF1 positivity, ×100. **(H)** Loss of membranous E-cadherin expression, ×100. **(I)** CD56 positivity, ×100. **(J)** CD10 positivity, ×100. Scale bars: 200 μm in **(A, C–J)**; 100 μm in B. **(K)** Macroscopic view of the resected surgical specimen including the sectioned pancreatic mass, the spleen, and peripancreatic fatty tissue. **(L)** Gross appearance of the intact spleen and surrounding peripancreatic fatty tissue. **(M)** Gross cross-section of the tumor showing a gray-red cut surface with a mixed solid-cystic appearance, hemorrhage, and focal necrosis.

Immunohistochemistry showed weak focal positivity for pan-cytokeratin (PCK), positivity for CD56, scattered positivity for synaptophysin (Syn), negativity for chromogranin A (CgA) and insulinoma-associated protein 1 (INSM1), positivity for vimentin and CD10, weak positivity for transcription factor E3 (TFE3), partial positivity for lymphoid enhancer-binding factor 1 (LEF1), nuclear and cytoplasmic positivity for beta-catenin, loss of membranous E-cadherin, and a Ki-67 labeling index of approximately 2% (**Figure 2**). Taken together with the histologic morphology, these findings supported the final diagnosis of pancreatic SPN.

### 2.7 Postoperative course and follow-up

The postoperative course was uneventful. Drain amylase levels were not elevated, the drainage tubes were removed without difficulty, and no clinically significant hemorrhage, infection, or pancreatic fistula occurred. Early postoperative CT showed expected changes after distal pancreatectomy with splenectomy. At approximately 1-month follow-up, contrast-enhanced CT demonstrated decreased postoperative fluid collection and no radiologic evidence of recurrence. At approximately 6-month follow-up, non-contrast abdominal CT demonstrated postoperative changes after resection of the pancreatic body-tail lesion and splenectomy; only slight blurring of the operative-field fat planes or minimal exudative change was described, without retroperitoneal lymphadenopathy, biliary dilatation, bowel dilatation, or a definite recurrent mass (**Figures 1H, I**). Continued radiologic follow-up was recommended. The patient’s diagnostic, therapeutic, and follow-up course is summarized in **Table 1** according to the CARE Guidelines.

**Table 1:** Patient timeline according to the CARE Guidelines.

Time point	Clinical event	Key findings and management
24 hours before admission	Symptom onset	The patient developed intermittent dull abdominal pain after strenuous physical activity, without nausea, vomiting, chest discomfort, or palpitations.
Admission	Initial clinical assessment	Physical examination showed mild upper abdominal tenderness without rebound tenderness or a clearly palpable mass. Past medical, surgical, and family histories were unremarkable.
Admission work-up	Laboratory examination	Routine blood tests, liver and renal function tests, coagulation parameters, and tumor markers were within normal limits.
Preoperative imaging	Computed tomography, computed tomography angiography/venography, and contrast-enhanced magnetic resonance imaging	Imaging localized a giant mixed solid-cystic lesion to the pancreatic body and tail. The lesion displaced adjacent organs, and the splenic vein was focally narrowed with collateral venous communication.
Preoperative multidisciplinary assessment	Diagnostic and operative planning	Solid pseudopapillary neoplasm was considered the leading preoperative diagnosis. The working differential diagnoses included pancreatic neuroendocrine tumor, pancreatoblastoma, mucinous cystic neoplasm, and accessory spleen-related lesion. Surgical planning prioritized complete tumor removal, avoidance of tumor rupture, and safe bleeding control.
Operation	Laparoscopic distal pancreatectomy with splenectomy	A giant tumor was found in the pancreatic body and tail. Because the tumor was densely adherent to the splenic vessels and spleen preservation was considered unsafe, laparoscopic distal pancreatectomy with splenectomy was performed.
Postoperative pathology	Final diagnosis	Histopathology confirmed pancreatic solid pseudopapillary neoplasm with negative surgical margins and no lymph node metastasis. Well-differentiated ectopic splenic tissue was identified in the peripancreatic fat.
Early postoperative period	Recovery	The postoperative course was uneventful. No clinically significant hemorrhage, infection, or pancreatic fistula occurred.
Approximately 1-month follow-up	Radiologic follow-up	Contrast-enhanced computed tomography showed decreased postoperative fluid collection and no radiologic evidence of recurrence.
Approximately 6-month follow-up	Radiologic follow-up	Non-contrast abdominal computed tomography showed postoperative changes after distal pancreatectomy and splenectomy, without a definite recurrent mass. Continued radiologic follow-up was recommended.

## 3 Discussion

### 3.1 Epidemiology and clinical presentation

SPN is a rare pancreatic tumor that predominantly affects adolescent girls and young women (1–4). Most cases grow slowly and present with subtle or nonspecific symptoms, such as abdominal discomfort, abdominal pain, or a palpable mass. With the wider use of imaging, incidental detection has become increasingly common (2–4). Although its overall malignant potential is low, large tumors may be associated with intratumoral hemorrhage, necrosis, and cystic degeneration, and rare cases may present with acute abdominal pain after trauma or spontaneous rupture (4, 6). In the present case, acute abdominal pain developed after strenuous activity, which was considered most likely related to intratumoral degeneration, hemorrhagic change, and altered local tension within a giant lesion after review of imaging and pathological findings. SPN should therefore remain an important differential diagnosis in young female patients presenting with a giant mixed solid-cystic pancreatic mass, especially when acute abdominal pain is present (2, 4, 5).

### 3.2 Pathogenesis and molecular pathology

The exact cell of origin of SPN remains incompletely understood, but aberrant activation of the Wnt/beta-catenin pathway is regarded as the central molecular event (7, 8). Most SPNs harbor CTNNB1 exon 3 alterations, which impair beta-catenin degradation, promote cytoplasmic accumulation, and drive nuclear translocation with downstream transcriptional activation (7, 8). Immunohistochemically, nuclear/cytoplasmic beta-catenin positivity together with loss of membranous E-cadherin constitutes the most characteristic diagnostic pattern (9–11). Partial LEF1 positivity in the present case provided additional support for this pathway abnormality (12).

The marked predominance of SPN in young women suggests that hormone-related signaling may also contribute to tumor development. Prior studies have shown expression of progesterone receptor and other hormone-related markers in a subset of SPNs, and a recent review further summarized potential interactions among progesterone receptor, estrogen receptor beta, androgen receptor, Wnt/beta-catenin signaling, Notch signaling, Hedgehog signaling, and epithelial-mesenchymal transition (4, 7, 13, 14). However, the biological and therapeutic significance of these findings remains incompletely defined, particularly in localized resectable tumors. Thyroid function tests in this patient were within normal ranges, including free triiodothyronine 5.03 pmol/L, free thyroxine 17.42 pmol/L, thyroid-stimulating hormone 2.339 μIU/mL, and thyroglobulin antibody 0.10 IU/mL. In this patient, serum sex hormones, including progesterone, estrogen, and androgen, were not measured because there were no clinical endocrine manifestations, and PR/ER/AR immunostaining was not included in the initial immunohistochemical diagnostic panel. This represents a limitation of the present report. If recurrent, metastatic, or unresectable disease is encountered, assessment of hormone receptor status may be useful for multidisciplinary discussion of individualized endocrine or targeted treatment strategies. Notably, despite a tumor diameter greater than 10 cm, the Ki-67 index in this case was only approximately 2%, in keeping with the relatively low proliferative activity and generally indolent clinical behavior of SPN (3, 4, 15).

### 3.3 Imaging diagnosis and differential diagnosis

SPN typically appears as a well-circumscribed mixed solid-cystic mass and may be associated with a capsule, hemorrhage, and necrosis (4, 5). CT is useful for lesion detection and assessment of compression of adjacent organs, whereas MRI is superior for demonstrating hemorrhagic components, solid-cystic architecture, and capsule-like features (5). In this patient, the preoperative MRI findings were highly consistent with the typical imaging appearance of SPN (**Table 2**). In giant lesions, however, the extent of cystic degeneration and hemorrhagic necrosis may overlap with mucinous cystic neoplasms, PanNETs, or pancreatoblastoma, thereby increasing the difficulty of precise preoperative characterization (4, 5).

**Table 2:** Imaging characteristics of solid pseudopapillary neoplasm (SPN) of the pancreas.

Modality	Typical findings	Diagnostic advantage
Unenhanced CT	Usually presents as a relatively well-circumscribed mixed solid-cystic mass with heterogeneous density, sometimes accompanied by cystic degeneration, hemorrhage, or necrosis	Useful for lesion detection and for assessing tumor size, location, and its relationship with adjacent organs
Contrast-enhanced CT	The solid component usually shows heterogeneous enhancement, often with a progressive enhancement pattern, while cystic and necrotic areas remain non-enhancing	Helpful for evaluating vascularity and for preliminary differentiation from other pancreatic tumors such as PanNET; also useful for assessing vascular compression
MRI	T1-weighted images usually show predominantly low signal intensity, with patchy high signal in hemorrhagic areas; T2-weighted images show heterogeneous mildly high signal, with higher signal in cystic areas; a capsule-like low-signal rim may be visible; the solid component typically shows progressive heterogeneous enhancement	More sensitive than CT for demonstrating intratumoral hemorrhage, mixed solid-cystic architecture, and capsule-like features, and is therefore more helpful for the preoperative characterization of SPN

PanNETs often show stronger arterial-phase enhancement, whereas SPN more commonly demonstrates progressive heterogeneous enhancement; however, this distinction is not absolute in giant tumors with necrosis, hemorrhage, and altered local hemodynamics (4, 5). Pancreatoblastoma is more typical in younger children, tends to be more aggressive, and is often associated with elevated AFP. Accessory spleen-related lesions are usually small nodules with enhancement behavior similar to that of normal splenic tissue (16, 17) (**Table 3**). Preoperative evaluation of a giant mixed solid-cystic lesion in the pancreatic body-tail region should therefore integrate patient age, sex, enhancement pattern, solid-cystic composition, evidence of hemorrhage, laboratory findings, and whether enhancement parallels that of the spleen (5, 16, 17).

**Table 3:** Imaging differential diagnosis of solid pseudopapillary neoplasm (SPN) and other pancreatic lesions.

Disease	Typical population/clinical background	Main imaging features	Key points for differentiation from SPN
SPN	Predominantly affects adolescents and young women	Well-circumscribed mixed solid-cystic mass, often with a capsule, hemorrhage, and necrosis; enhancement is usually progressive and heterogeneous	Diagnosis is supported by age, sex, mixed solid-cystic architecture, hemorrhagic features, and progressive enhancement
Pancreatic neuroendocrine tumor (PanNET)	More common in adults; may be associated with functional syndromes	Usually a solid mass, sometimes with cystic degeneration or necrosis, often showing marked arterial-phase enhancement	PanNET is typically more hypervascular with stronger arterial enhancement; SPN more often shows mixed solid-cystic architecture, hemorrhage, and progressive heterogeneous enhancement
Pancreatoblastoma	More common in younger children	Often a large mass, either solid or mixed solid-cystic, with more aggressive behavior	Younger age, greater aggressiveness, and frequent AFP elevation help distinguish it from SPN
Mucinous cystic neoplasm (MCN)	More common in middle-aged women	Predominantly cystic lesion, often with septations and cyst wall changes, usually with less solid component	SPN more often presents as a mixed solid-cystic mass with hemorrhagic degeneration and necrosis, whereas MCN is more predominantly cystic
Accessory spleen-related lesion/intrapancreatic accessory spleen	May occur at any age, usually without specific symptoms	Usually a small solid nodule in the pancreatic tail with enhancement behavior similar to normal splenic parenchyma	These lesions are usually small and enhance synchronously with the spleen; SPN is often larger, more complex, and associated with cystic change and hemorrhage

### 3.4 Pathology and immunohistochemical features

The characteristic histologic appearance of SPN consists of mixed solid areas and pseudopapillary structures, often accompanied by cystic change, hemorrhage, and necrosis (7, 9, 18). Pathological diagnosis depends not only on morphology but also on a highly informative immunohistochemical profile. Nuclear/cytoplasmic beta-catenin positivity and loss of membranous E-cadherin are the most distinctive features, while CD10 and vimentin are commonly positive and CgA is usually negative or only focally weak (9–12, 18). SPN may also show focal Syn or CD56 expression, and it should not be classified as PanNET on the basis of a single neuroendocrine marker alone (11, 18). In the present case, negative CgA, only scattered Syn positivity, positive CD10 and vimentin, CD56 positivity, partial LEF1 positivity, and the beta-catenin/E-cadherin pattern together were sufficient to support the diagnosis of SPN. Recent evidence also supports LEF1 and related Wnt-pathway markers as useful adjuncts in diagnostically challenging or pediatric cases, but they should complement rather than replace the core beta-catenin/E-cadherin evidence chain (12, 19) (**Table 4**).

**Table 4:** Key histologic and immunohistochemical features of solid pseudopapillary neoplasm of the pancreas.

Category	Marker/item	Typical pattern	Diagnostic significance
Morphology	Architecture	Mixed solid and pseudopapillary structures, often accompanied by hemorrhage, necrosis, and cystic degeneration	Provides the basic histologic framework for the diagnosis of SPN
Core marker	β-catenin	Nuclear/cytoplasmic positivity	Indicates activation of the Wnt/β-catenin pathway and is one of the most informative diagnostic markers
Core marker	E-cadherin	Loss of membranous staining	Forms a highly characteristic diagnostic pair with aberrant β-catenin localization
Supportive marker	CD10, vimentin	Usually positive	Supports the diagnosis of SPN, especially in the differential diagnosis from PanNET
Neuroendocrine markers	Syn, CD56, CgA	Syn/CD56 may be focally positive, whereas CgA is usually negative	Helps avoid misdiagnosing SPN as PanNET based on isolated neuroendocrine marker positivity

### 3.5 Treatment strategy

Complete resection with negative margins remains the core therapeutic principle for SPN (3, 4). Given the generally favorable prognosis, organ and functional preservation should be considered whenever anatomically feasible, and spleen-preserving distal pancreatectomy may be preferred for body-tail lesions when safe dissection is possible, particularly to reduce the long-term infectious burden associated with asplenia (3, 4, 20, 21). In the present case, however, the tumor was giant, the splenic vein was narrowed with collateral circulation, and dense adhesion to the splenic vessels was encountered intraoperatively. Under these circumstances, spleen-preserving dissection carried a substantial risk of bleeding and tumor rupture, and combined splenectomy was more consistent with operative safety and oncologic principles (3, 4).

In recent years, local therapies such as endoscopic ultrasound-guided radiofrequency ablation have been explored for small, highly selected SPN cases, and isolated reports have also described endocrine therapy, targeted therapy, or interventional therapy in recurrent, metastatic, or unresectable disease (13, 22–25). However, the current evidence comes mainly from case reports and small series and remains insufficient to challenge the dominant role of surgery in resectable SPN (3, 4, 13, 22–25). For a giant but resectable lesion such as the present case, formal surgical resection remains the optimal choice (3, 4) (**Table 5**).

**Table 5:** Summary of management strategies for solid pseudopapillary neoplasm of the pancreas.

Strategy	Clinical setting	Key point	Evidence position
Standard surgery	Resectable SPN	Complete resection with negative margins (R0 resection)	Current first-line treatment with the strongest evidence base
Organ preservation	Favorable anatomy with safe vascular dissection	Spleen-preserving distal pancreatectomy may be considered	Appropriate in selected cases, provided that rupture and bleeding risks are not increased
Combined splenectomy	Giant tumors, splenic vessel adhesion or compression, collateral circulation	Formal distal pancreatectomy with splenectomy	More appropriate when spleen preservation cannot be achieved safely
Local ablation	Small, highly selected lesions or patients unfit for surgery	Endoscopic ultrasound-guided radiofrequency ablation	Reported in case reports or small series; not a replacement for surgery
Systemic/individualized therapy	Metastatic, recurrent, or unresectable disease	Everolimus, endocrine therapy, or molecularly targeted therapy	Supported mainly by case reports and should be individualized in multidisciplinary discussion

### 3.6 Prognosis and follow-up

The overall prognosis after complete resection of SPN is favorable, but a minority of patients may still develop local recurrence, peritoneal seeding, or distant metastasis (2–4, 6, 26, 27). Reported risk factors include larger tumor size, lymphovascular invasion, positive margins, higher Ki-67 index, synchronous metastasis, and tumor rupture (6, 15, 26, 27). In this patient, the margins were negative, the lymph node was negative, and Ki-67 was low, indicating relatively low risk; however, the maximum tumor diameter of 11 cm still supports the need for long-term radiologic surveillance. For giant SPN, a pragmatic follow-up strategy is to consider CT or MRI every 6 months during the first 2 years and annually thereafter for at least 5 years, with longer follow-up when high-risk features are present. Long-term infectious risk after splenectomy should also not be underestimated (20, 21, 28). Postoperative care for adolescent patients undergoing splenectomy should include structured infection-prevention counseling, pneumococcal, meningococcal, annual influenza, and Haemophilus influenzae type b vaccination according to age and local policy, individualized antibiotic prophylaxis when appropriate, standby emergency antibiotics when timely medical care may not be available, and clear instructions that fever in an asplenic patient requires urgent medical assessment (20, 21, 28).

## 4 Conclusion

This case shows that, although giant SPN is generally a low-grade malignant tumor with favorable overall prognosis, it may present with acute abdominal pain in the setting of intratumoral degeneration, hemorrhagic necrosis, and local vascular compression. MRI is valuable for diagnosis and differential diagnosis, while nuclear/cytoplasmic beta-catenin positivity combined with loss of membranous E-cadherin provides a reliable pathological evidence chain. Complete resection remains the core treatment principle; in selected giant SPNs with splenic vessel narrowing, collateral circulation, and dense adhesion to splenic vessels, distal pancreatectomy with splenectomy may be a reasonable and safer option. Beyond oncologic surveillance, long-term infection prevention after splenectomy should also be emphasized.

## Data Availability

The raw data supporting the conclusions of this article will be made available by the authors, without undue reservation.
